# Cellular and Hormonal Disruption of Fetal Testis Development in Sheep Reared on Pasture Treated with Sewage Sludge

**DOI:** 10.1289/ehp.8028

**Published:** 2005-07-11

**Authors:** Catriona Paul, Stewart M. Rhind, Carol E. Kyle, Hayley Scott, Chris McKinnell, Richard M. Sharpe

**Affiliations:** 1 MRC Human Reproductive Sciences Unit, Centre for Reproductive Biology, Queen’s Medical Research Institute, University of Edinburgh, Edinburgh, United Kingdom; 2 Macaulay Institute, Craigiebuckler, Aberdeen, United Kingdom

**Keywords:** anti-Müllerian hormone, environmental chemicals, follicle-stimulating hormone, FSH, gonocyte, inhibin-A, Leydig cell, LH, peritubular myoid cell, Sertoli cell, sewage sludge, testosterone

## Abstract

The purpose of this study was to evaluate whether experimental exposure of pregnant sheep to a mixture of environmental chemicals added to pasture as sewage sludge (*n* = 9 treated animals) exerted effects on fetal testis development or function; application of sewage sludge was undertaken so as to maximize exposure of the ewes to its contents. Control ewes (*n* = 9) were reared on pasture treated with an equivalent amount of inorganic nitrogenous fertilizer. Treatment had no effect on body weight of ewes, but it reduced body weight by 12–15% in male (*n* = 12) and female (*n* = 8) fetuses on gestation day 110. In treated male fetuses (*n* = 11), testis weight was significantly reduced (32%), as were the numbers of Sertoli cells (34% reduction), Leydig cells (37% reduction), and gonocytes (44% reduction), compared with control fetuses (*n* = 8). Fetal blood levels of testosterone and inhibin A were also reduced (36% and 38%, respectively) in treated compared with control fetuses, whereas blood levels of luteinizing hormone and follicle-stimulating hormone were unchanged. Based on immunoexpression of anti-Müllerian hormone, cytochrome P450 side chain cleavage enzyme, and Leydig cell cytoplasmic volume, we conclude that the hormone changes in treated male fetuses probably result from the reduction in somatic cell numbers. This reduction could result from fetal growth restriction in male fetuses and/or from the lowered testosterone action; reduced immunoexpression of α-smooth muscle actin in peritubular cells and of androgen receptor in testes of treated animals supports the latter possibility. These findings indicate that exposure of the developing male sheep fetus to real-world mixtures of environmental chemicals can result in major attenuation of testicular development and hormonal function, which may have consequences in adulthood.

There is growing concern that exposure of adults and, especially, of the developing fetus to environmental chemicals could have potentially detrimental effects on various aspects of health, particularly reproductive development. This concern stems from four areas of information. First, evidence for a range of reproductive disorders in various wildlife species, ranging from aquatic snails and fish to mammals, has established close associations with environmental chemical exposure [International Programme on Chemical Safety (IPCS) 2002; Toppari et al. 1996]. Second, the occurrence and possibly increasing prevalence of reproductive disorders in human males are thought to originate in fetal life (Sharpe and Skakkebaek 2003; Skakkebaek et al. 2001) and can be induced in animal models by fetal exposure to environmental chemicals (Fisher et al. 2003; Mylchreest et al. 1999; Parks et al. 2000). Third, our understanding is growing regarding the many pathways by which particular environmental chemicals—so-called endocrine disruptors (EDs)—can alter hormone production, metabolism, and/or action in animals or in isolated cells (IPCS 2002). Fourth, evidence shows that humans are exposed to a broad range of environmental chemicals [Centers for Disease Control and Prevention (CDC) 2003].

In contrast to some of the reproductive disorders in wildlife, in which an environmental chemical cause is clearly established (IPCS 2002), concerns for human reproductive health are based largely on extrapolation from laboratory animal studies in which exposure to supraenvironmental levels of test chemicals have been used (IPCS 2002; Sharpe and Irvine 2004). It can be argued that, because induction of adverse effects in such models only occurs at levels of exposure considerably greater than those experienced by humans, effects at real-world concentrations of the chemical in question are unlikely to occur. A counterargument is that, in normal life, humans (and wildlife) are exposed to a cocktail of environmental chemicals (CDC 2003) that may have additive or interactive hormonal effects (Rajapakse et al. 2002). In some areas of toxicology, understanding of the toxicologic effects of mixtures of environmental chemicals is relatively well developed and can be modeled (see Liao et al. 2002). In contrast, for EDs or other environmental chemicals with potential effects on the developing reproductive system, only a small number of studies in laboratory settings have been undertaken (Rajapakse et al. 2002; Tindall and Ashby 2004). To date, such studies have established only proof of principle (Rajapakse et al. 2002) and studies *in vivo* in laboratory animals are at a similarly early stage of development (Tindall and Ashby 2004). Moreover, such studies are confined to combinations of a small number of selected test chemicals rather than reflecting the multitude of environmental chemical exposures to which humans are subjected in the course of a normal day. Although there are major difficulties in establishing whether such exposures might contribute to human disorders, three developments have given them a new urgency: *a*) the evidence for increasing prevalence of human male reproductive disorders as a consequence of testicular maldevelopment in fetal life (Sharpe and Skakkebaek 2003; Skakkebaek et al. 2001); *b*) the induction of comparable effects in laboratory animals as a result of fetal exposure to certain phthalate esters (Fisher et al. 2003; Mylchreest et al. 1999; Parks et al. 2000); and *c*) recent evidence that associates human fetal exposure to phthalates with reduced production/action of androgens in the male fetus (Swan et al. 2005). Collectively, these studies highlight the potential vulnerability of the male fetus to endocrine disruption as a result of environmental chemical exposures.

One situation with potential fetal exposure to a naturally occurring mixture of environmental chemicals is in farm animals that are reared on pasture that has been fertilized with sewage sludge. The latter is broadly reflective of most human chemical exposures in that it contains outputs from domestic, agricultural, and industrial sources and contains a cocktail of man-made chemicals (phthalates, alklyphenolics, bisphenolics, polyaromatic hydrocarbons, organochlorine pesticides, etc.) as well as inorganic compounds and heavy metals such as lead and mercury (Smith 1995; Webber and Lesage 1989). Land application of sewage sludge is a technique widely used around the world and has grown in popularity due to tightening of regulations for disposal of such waste at sea and the pressures to recycle waste (Commission of the European Communities 1994; Swanson et al. 2004). In the present studies, we have investigated whether long-term exposure of ewes to the mix of chemicals present in sewage sludge affects development of the fetal testis when these animals become pregnant. Using a protocol that was designed to maximize potential exposure to the chemicals present in sewage sludge, we have evaluated the development of Sertoli and Leydig cells in terms of their numbers and hormonal function as well as germ cell numbers.

## Materials and Methods

### Animals, blood, and tissue collection.

Animals were maintained on pasture at the Macaulay Institute research station at Hartwood, Lanarkshire, Scotland. Animals were maintained at conventional stocking rates, according to the pasture height. They were inspected by a qualified shepherd on a daily basis, and routine animal care and vaccination procedures were conducted, as prescribed by best practice protocols.

From July 1997 until the end of July 1999, liquid, digested sewage sludge was applied twice annually to three 9-ha plots until five separate applications had been made. Thereafter, thermally dried sludge pellets were applied twice annually at similar rates because of changes in sludge production and spreading practices by the U.K. water authorities at the time. The composition of the sludge on a dry-matter basis was not altered. On each occasion, sludge was applied at a rate of 2.25 metric tons of dry matter per hectare to the whole of each plot, using either a pivot irrigation system (liquid sludge; estimated to cover > 95% of the surface) or a lime spreader (pelleted dried sludge), respectively. This rate of sludge application, which resulted in the application of about 225 kg nitrogen/ha/year, was consistent with normal management practice at the time, although recommendations for good practice in the United Kingdom have since been revised (SEDE and Arthur Andersen 2001). Our studies were designed to result in the maximum rate of contamination of the herbage and topsoil, and thus the maximum likely risk of exposure of grazing animals to the chemical constituents of sewage sludge through their food. Animals were not allowed to graze the pasture for a minimum of 3 weeks after sludge application, as prescribed by legislation (Great Britain Parliament 1989). Control ewes were maintained on similar pasture to which 225 kg of nitrogen/ha/year was applied using conventional, inorganic fertilizers. In the study flocks, treated and control groups consisted of three replicate groups of five breeding ewes in each of four age categories, so that at any one time there were 120 ewes in the study. All ewes were from similar genetic stock. Each year, ewes that were 6 years of age were slaughtered during pregnancy, and replacement animals were brought into the flocks to be bred for the first time. Stocking rates for ewes on control and sewage sludge-fertilized pastures were comparable. There was no supplementary feeding for either group.

Nine ewes maintained for the previous 5 years on conventionally fertilized pasture (controls) and nine ewes reared on sewage sludge-treated pasture were used for the present studies. All ewes were synchronized in estrus, using progestagen sponges, before mating to rams from the same genotype and source. Because estrus was synchronized, conception can be predicted to have occurred within a 48-hr window at the end of the first cycle after sponge removal. This was used as the basis for determining gestational age of approximately 110 days (GD110), when all animals were euthanized according to Schedule 1 protocols as defined by the U.K. Animals (Scientific Procedures) Act, 1986. Fetuses were collected from both sets of ewes, and all of the resulting males (*n* = 12 in both control and treated groups) were used for the studies detailed below. Maternal and fetal body weight and fetal testis weight were recorded at slaughter; fetal blood samples were also collected and serum isolated after centrifugation and stored at −20°C. Testes were fixed for 6 hr in Bouin’s fixative and then transferred to 70% ethanol until analysis. The testes were subsequently cut in half sagittally using a razor blade and processed in an automated Leica processor (Leica, Nussloch, Germany) before embedding in paraffin wax. Fetal body weight for female fetuses was also recorded.

### Immunohistochemistry.

Sagittal 5 μm sections of each fetal testis were cut, floated onto slides, and dried at 50°C overnight. Slides were dewaxed in xylene, hydrated gradually through graded alcohols, and washed in water. Some antibodies [androgen receptor (AR) and Ki-67] required antigen retrieval by pressure cooking in 0.01 M citrate buffer (pH 6.0), after which sections were washed in Tris-buffered saline (TBS; 0.05 M Tris-HCl, pH 7.4, 0.85% NaCl) twice for 5 min. Endogenous peroxidase activity was blocked by immersion in 3% (vol/vol) hydrogen peroxide in methanol (BDH Laboratory Supplies, Poole, Dorset, UK) for 30 min, followed by rinsing in tap water and washing in TBS for 5 min. Nonspecific binding sites were blocked with the appropriate normal serum diluted 1:5 in TBS containing 5% bovine serum albumin (BSA; Sigma Chemical Co., Poole, Dorset, UK) applied for 30 min at room temperature. For anti-Müllerian hormone (AMH) and α-smooth muscle actin (SMA) immunostaining, normal rabbit serum was used; for all other antibodies, normal swine serum was used (Scottish Antibody Production Unit, Carluke, Scotland, UK). Tissues were then incubated with the primary antibody (Table 1) diluted in the appropriate blocking serum, and incubated overnight in a humidified chamber at 4°C.

After washing twice in TBS for 5 min, secondary antibodies were added to sections and incubated for 30 min at room temperature. A 1:500 dilution in the appropriate blocking serum of biotinylated rabbit anti-mouse IgG (DAKO, High Wycombe, UK) for SMA and Ki-67, biotinylated rabbit anti-goat IgG (DAKO) for AMH, and biotinylated swine anti-rabbit IgG (DAKO) for AR and P450 side chain cleavage enzyme (P450scc) was used. After two 5-min washes in TBS, the biotinylated antibody was linked to horseradish peroxidase (HRP) by 30 min incubation with avidin-biotin-HRP complex (ABC-HRP; DAKO) diluted in Tris-HCl (pH 7.4). This was followed by two 5-min washes in TBS. Antibody localization was determined by application of liquid diaminobenzidine substrate chromogen system (DAKO) for 1–5 min until the brown positive staining in control sections was optimal as determined by repeated microscopic examination. The color reaction was stopped by immersion in water. Sections were subsequently counterstained with hematoxylin, dehydrated in graded ethanols, cleared in Histoclear (VWR, Lutterworth, UK) and then xylene, and mounted using Pertex mounting medium (CellPath plc, Hemel Hempstead, UK). Testis sections from all control and treated animals were run for each antibody at the same time and under the same conditions to ensure comparability. Slides were then evaluated subjectively but systematically so that the intensity of immunostaining was scored for each animal on a scale from negative (−) through weakly (+) to strongly (++++) positive. Median values for immunostaining score were then determined for control (C) and treated (T) animals and compared. Immunostained sections were photographed using a Provis microscope (Olympus Optical, London, UK) fitted with a digital camera (Canon EOS 10D, Surrey, UK). Captured images were then transferred to a personal computer and compiled using Photoshop 7.0 (Adobe Systems Inc., San Jose, CA, USA).

### Determination of gonocyte, Sertoli, and Leydig cell numbers per testis.

Stereologic analyses to determine cell numbers used AMH-immunostained slides for Sertoli cell and gonocyte counts and P450scc-immunostained slides for Leydig cell counts. For measurements, images were captured from an Olympus BH2 microscope using a video camera (Hitachi HV-C20, Tokyo, Japan) and were analyzed with Image Pro Plus software with a Stereology 5.0 plug-in (Media Cybernetics, Berkshire, UK). Slides were viewed using a 40× objective for counting and a 100× oil-immersion objective for nuclear volume measurement. The software was used to trace around each section, creating an area of interest (AOI). Forty fields, randomly selected by the program, within the AOI were then examined on one sagittal cross-section of the testis from each animal. A grid consisting of 432 evenly distributed points was superimposed over the digital image of each microscopic field studied. The numbers of intersections on the grid overlying the component of interest (Sertoli cell nuclei, Leydig cell nuclei, and cytoplasm or gonocyte nuclei) were then counted using a manual tag system. The ratio of the total count per testis to the total number of points possible (40 fields × 432 points per field) multiplied by testicular volume (cubic centimeters, derived from testis weight) was considered to be the absolute volume of the cellular component in question. These data were converted to absolute numbers of cells per testis by determining the average volume of the nucleus of each cell type. The mean nuclear volume of Leydig cells and gonocytes was measured using the stereology program, which calculates volume based on the assumption that all objects are spherical. The software takes the average of three measured diameters per nucleus. A minimum of 70 nuclei per cell type per testis were measured, and an average nuclear volume was then calculated. Because the Sertoli cell nucleus is not spherical, its volume was determined using a different approach (Mahood et al. 2005; Sharpe et al. 2000). An AOI was created by drawing around the Sertoli cell nucleus, within which the computer program then determined the average length of several diameters measured at 2° intervals that passed through the center of the nucleus. This was measured for a minimum of 70 Sertoli cell nuclei per testis, and mean nuclear volume was then determined.

Fetal ovine testes possess a fairly thick tunica (capsule) and contain a large central rete, neither of which contain Sertoli and Leydig cells or gonocytes. Because estimation of cell number per testis used testis weight as the measure of overall testis volume, this would lead to overestimation of cell numbers. To overcome this problem, the percentage of testis volume occupied by rete and capsule was measured using the Image Pro Plus software. One representative fetal testis from a sewage-sludge–exposed mother and one from a control mother was serially sectioned, and every 30th slide was immunostained for AMH, which does not stain the rete or the capsule and therefore clearly demarcates these structures. Three areas were then measured in the same way that the nuclear area of Sertoli cells was measured, described above: the rete, the testicular parenchyma (including rete), and the entire testis. The average percentage of testicular volume occupied by parenchyma was then calculated from these measurements, and this percentage used to correct the average volume of the testis in each animal before calculation of cell number per testis.

### Determination of the Sertoli cell and gonocyte proliferation index.

The proliferation index of Sertoli cells was determined by counting the number of Ki-67–immunopositive and immunonegative Sertoli cell nuclei in 30 seminiferous cords per animal. The proliferation index of gonocytes was calculated in the same way. The Leydig cell proliferation index was not determined because Ki-67–immunopositive Leydig cells could not be reliably distinguished from other interstitial cells.

### Hormone measurements.

Fetal serum levels of follicle-stimulating hormone (FSH) and luteinizing hormone (LH) were measured by radioimmunoassays that have been described and validated previously for sheep (McNeilly et al. 1986); the assay standards used were NIDDK-FSH-RP2 and NIH-LS18, and assay sensitivities were 0.1 and 0.2 ng/mL for FSH and LH, respectively. Serum levels of testosterone were measured using an enzyme-linked immunosorbent assay adapted from an earlier radioimmunoassay method (Corker and Davidson 1981), as described previously (Rivas et al. 2002); the limit of detection was 8 pg/mL. Serum levels of inhibin A, the main inhibin type produced by Sertoli cells in the male sheep (McNeilly et al. 2002), were measured using a two-site, enzyme-linked immunoassay that uses a capture antibody directed against amino acid sequence 82–114 of the human and ovine βA subunit and a C-specific biotinylated monoclonal antibody raised against a synthetic peptide that corresponds to amino acid sequence 1–32 of the human α-C subunit, as the detection antibody (Knight et al. 1998); the limit of detection was 20 pg/mL. For each hormone, all fetal blood samples were run in a single assay.

### Statistical analysis.

We compared weights, cell numbers, and hormone levels in control and treated animals using Student’s *t*-test. Correlations between variables were assessed using the Pearson correlation test (Graphpad software, San Diego, CA, USA). In the present studies, four C and six T male fetuses were derived from multiple pregnancies and might therefore be viewed as not being truly independent samples. When values for such co-twins were meaned, and these values then reentered in statistical tests, it did reduce the level of significance for all parameters, but not so as to alter any of the main conclusions of the study. Mean values for all parameters for these co-twins also did not differ significantly from the remaining animals. Data are therefore presented with co-twins included as independent samples.

## Results

Testes taken from male fetuses from ewes grazed on sewage-sludge–treated pastures are referred to as T testes, and those taken from male fetuses from ewes maintained on conventionally fertilized pasture are referred to as C testes. Two of the control ewes are known to have escaped into adjoining pastures in which a ram was present, between progestagen sponge removal and the prescribed mating date. Because the size of the four fetuses from these animals (F22, 23, 39, 40; Table 2) was entirely consistent with them having been conceived one cycle (17 days) earlier than were the remaining fetuses in the study, on the basis of known growth trajectories, it was concluded that they were probably mated one cycle earlier than the remaining animals. Similarly, the size of the T animal F47 (Table 2) indicated that it was probably conceived one cycle later than the remaining animals, based on fetal weight. Exclusion of these five animals from analyses did not materially affect the results or conclusions from these studies, although it did reduce the statistical power and significance of several of the findings and, in the instance of the most variable parameter (testosterone levels), it resulted in loss of statistical significance. For completeness, data pertaining to the five fetuses conceived at different times are indicated in all of the figures, and *p*-values are presented both with and without inclusion of data from these fetuses. Similarly, in the text below, where percent reductions caused by treatment are referred to, two values are quoted, the first excluding the five fetuses and the second including them.

### Effects of treatment on maternal and fetal body weight and fetal testis weight.

Maternal exposure of ewes to pastures treated with sewage sludge (for the preceding 5 years, including the gestational period of 110 days) resulted in a 15–36% reduction in body weight and a 32–46% reduction in testis weight of male fetuses compared with those of C fetuses, although maternal body weight was similar in the two groups (Table 2). The mean (± SD) body weight of female fetuses from the same treated ewes (1,400 ± 183 g; *n* = 8) was also significantly reduced (*p* < 0.05) versus controls (1,593 ± 258 g; *n* = 15). Mean body weights were significantly higher (*p* < 0.05) in male C fetuses than in female C fetuses, but this sex difference was minimal in T animals. A significant positive correlation was observed between testis weight and body weight in both T (*p* < 0.005, *r* = 0.76) and C (*p* < 0.0001, *r* = 0.92) male fetuses, although this ratio was at least 20% lower in T than in C animals (Table 2).

### Cell number per testis.

**Figure 1:**
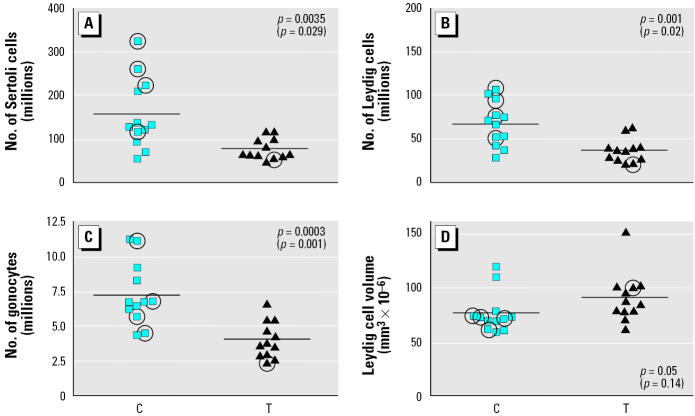
Numbers of Sertoli cells (*A*), Leydig cells (*B*), gonocytes (*C*), and Leydig cell cytoplasmic volume (*D*) in the testes of C and T fetuses on GD110. Each symbol corresponds to the cell number for an individual C or T animal (*n* = 12 per group); symbols that are circled indicate animals for which the gestational age may be different from GD110, and *p*-values in parentheses indicate the effect of removing these animals from statistical analysis. The horizontal line indicates the mean for each group.

The number of Sertoli cells per T testis was 34–51% lower than in C testes at GD110 (*p* < 0.05; Figure 1A). However, average Sertoli cell nuclear volume remained relatively constant in the T (95.4 ± 8.8 μm^3^) and C (100 ± 10.5 μm^3^) testes.

The number of Leydig cells per testis was reduced by 37–46% in T compared with C testes (Figure 1B). The mean nuclear volume per Leydig cell for T testes (122 ± 14.7 μm^3^), however, was similar to that for controls (112 ± 14.5 μm^3^). The cytoplasmic volume of Leydig cells was comparable in T (91.4 ± 23.0 × 10^−6^ μm^3^) and C testes (79.6 ± 18.7 × 10^−6^ μm^3^) (Figure 1D), implying that the steroidogenic function of individual Leydig cells in T testes is not impaired.

As with Sertoli and Leydig cells, the total number of gonocytes per testis was significantly (*p* = 0.001) reduced (by 43–44%) in T compared with C testes (Figure 1C). Again, there was no significant difference in mean nuclear volume per gonocyte between T (294.7 ± 32.7 × 10^−6^ μm^3^) and C (310.1 ± 29.1 × 10^−6^ μm^3^) testes. Because multinucleated gonocytes have been shown to be induced in the fetal rat testis by *in utero* exposure to certain phthalates (Fisher et al. 2003; Mylchreest et al. 1999), we searched for these in C and T testes, but only occasional such cells (one to five per testis cross-section) were found in both groups.

Despite the differences in absolute cell number, described above, there was no significant difference between the T and C testes in the number of Sertoli cells (respectively, 180.8 × 10^6^ cells/g and 193.5 × 10^6^ cells/g), Leydig cells (273.2 × 10^6^ cells/g and 279.9 × 10^6^ cells/g), or gonocytes (20.0 × 10^6^ cells/g and 21.2 × 10^6^ cells/g) when expressed per gram of testis. There was also no significant difference in the ratios of each of the aforementioned cell types with each other in C and T testes (data not shown).

### Changes in fetal plasma hormone levels.

**Figure 2:**
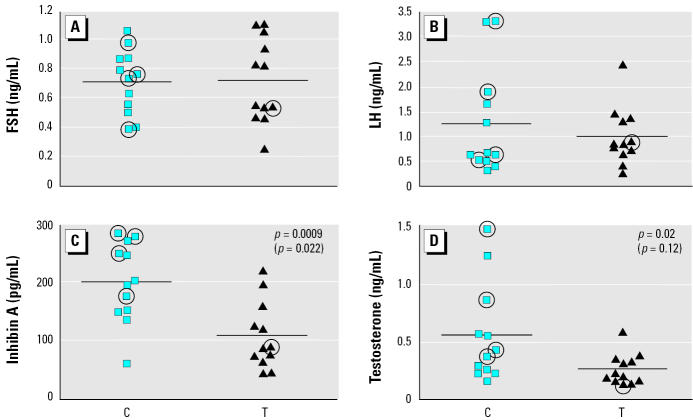
Plasma levels of FSH (*A*), LH (*B*), inhibin A (*C*), and testosterone (*D*) in the testes of C and T fetuses on GD110. Each symbol corresponds to the cell number for an individual C or T animal (*n* = 12 per group); symbols that are circled indicate animals for which the gestational age may be different from GD110, and *p*-values in parentheses indicate the effect of removing these animals from statistical analysis. The horizontal line indicates the mean for each group.

There was no significant difference in plasma levels of either FSH or LH between T and C animals on GD110 (Figure 2A,B). However, plasma inhibin A concentrations were significantly decreased, by 38–46%, in T animals (*p* < 0.05; Figure 2C). Plasma testosterone levels showed a reduction of 50% in T compared with C testes (*p* < 0.05), and although a decrease in mean levels was still evident (36%) when excluding the five animals referred to above, it was no longer statistically significant (Figure 2D). We applied Pearson’s correlation test to determine the correlation between plasma levels of FSH and inhibin A and between LH and testosterone, but we found no significant correlation in either case.

### Correlation between plasma hormone levels and cell number.

Because FSH can regulate Sertoli cell number and LH regulates testosterone secretion by Leydig cells, we explored the possible existence of significant correlations between these sets of parameters. No significant correlation between plasma FSH levels and Sertoli cell number or between LH and testosterone levels was found in either T or C animals (data not shown), implying that the lower Sertoli cell number and testosterone levels in T animals are not explained by altered gonadotropin levels.

### Cell proliferation (Ki-67 immunostaining) in seminiferous cords.

We determined the proliferation index for Sertoli cells and gonocytes on the basis of the percentage of cells immunostained for Ki-67. There was no significant difference in the proliferation index in T versus C testes for either Sertoli cells (T, 18.6 ± 4.3%; C, 19.1 ± 3.1%; mean ± SD) or gonocytes (T, 31.4 ± 6.2%; C, 32.3 ± 7.2%).

### Evaluation of testicular cell function using cell-specific and other markers.

#### Sertoli cells.

**Figure 3:**
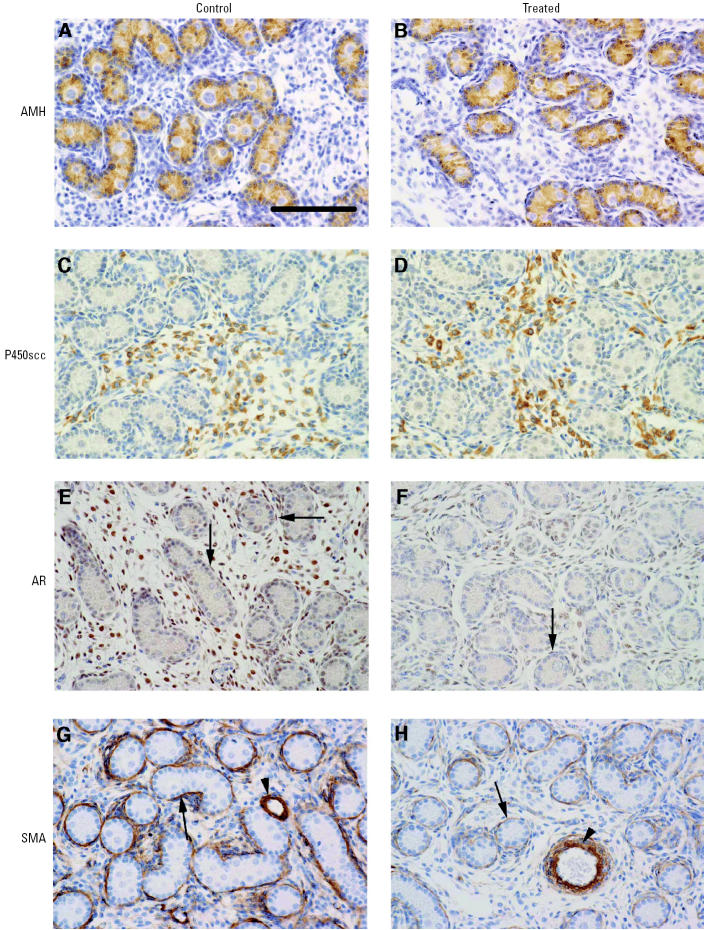
Immunoexpression (brown staining) of AMH (*A*, *B*), P450ssc (*C*, *D*), AR (*E*, *F*), and SMA (*G*, *H*) in testicular sections from representative C and T animals. Note the lower expression of AR (*E, F*) in interstitial and peritubular cells (arrows) and the lower expression of SMA (*G, H*) in peritubular cells (arrows) of T animals, compared with C animals. Arrowheads in (*G, H*) indicate SMA staining in perivascular cells. Bar = 100 μm.

In order to determine whether or not there were any treatment effects on Sertoli cell function, immunoexpression of AMH was evaluated and was found to be of similar intensity in T and C testes (Figure 3A,B).

#### Leydig cells.

For Leydig cell function, we evaluated immunoexpression of two protein markers: P450scc and 3β-hydroxysteroid dehydrogenase (3β-HSD). No difference in the intensity of immunoexpression of P450scc was observed between T and C testes (Figure 3C,D). Immunoexpression of 3β-HSD was of a very low intensity in both T and C testes and so was not considered to be a good means of Leydig cell identification or of evaluating their function.

#### AR immunoexpression.

Nuclear immunoexpression of the AR was evident in interstitial and peritubular cells whereas Sertoli cells were largely immunonegative. There appeared to be less intense immunostaining for AR in interstitial and peritubular cells in T compared with C testes (Figure 3E,F). There was also strong immunostaining of AR in the cells of the rete epithelium in testes from both control and treated animals (data not shown).

#### Peritubular myoid cells.

Seminiferous cords appeared to be normally formed in T and C testes, on gross inspection. However, mean testosterone levels were lower in T than C testes, so immunoexpression of SMA in peritubular cells was evaluated because it is suggested to be androgen regulated in some species (Schlatt et al. 1993). Immunostaining intensity was generally lower in T than in C testes (Figure 3G,H).

## Discussion

The principal aim of this study was to determine whether or not long-term experimental exposure of pregnant ewes to real-world cocktails of environmental chemicals (present in sewage sludge applied to the pasture) had any effect on testicular development in the male fetus. This was prompted by concerns about deteriorating human male reproductive health, in particular falling sperm counts (Swan et al 2000), and its possible relationship to environmental chemical exposures during fetal development (Sharpe and Irvine 2004; Sharpe and Skakkebaek 2003). Our results show that long-term exposure of breeding ewes to a mixture of chemicals added to pasture in sewage sludge (T animals), according to standard farming and European Union–recommended practices at the time the study was initiated (1997), resulted in major reductions (32–51%) in the numbers and hormonal function of the two principal somatic cell types of the fetal testis (Sertoli and Leydig cells) as well as a parallel reduction in the numbers of fetal germ cells. These changes were associated with growth restriction of male and female fetuses; body weight in the pregnant ewes was unaffected. The present study was restricted to late fetal life and therefore did not address whether the adverse testicular changes in T male fetuses have any permanent consequences. However, it might be anticipated that some aspects of the masculinization process in T male offspring could be attenuated (due to suppression of testosterone levels), as might sperm-producing capacity in adulthood (due to reduced Sertoli cell number). These remain to be explored, as does the more complex issue of which constituents of the treatment might have induced the adverse effects on testicular development. Under the present study conditions, sludge application has only minor effects on the soil concentrations of at least some of the more readily degraded EDs (Rhind et al. 2002); other studies indicate that the same is generally true of other, more persistent EDs (Smith 1995). It is therefore likely that the presently observed effects on the male fetus after exposure to sewage sludge reflects the effects of exposure to a mixture of environmental chemicals, and these may not be EDs as such.

In the present study we used established stereologic methods (Mahood et al. 2005; Sharpe et al. 2000) to quantify the total number of Sertoli cells, Leydig cells, and gonocytes per testis. There was a marked and consistent reduction (between 32 and 51%) in numbers of all three cell types in T compared with C fetal testes. The numerical changes in Sertoli and Leydig cells in treated animals were matched by parallel reductions in hormone production by these two cell types, as evidenced by the blood levels of inhibin A (secreted by Sertoli cells) and testosterone (secreted by Leydig cells), although the latter change was only statistically significant when all animals from the study were included. The similarity in degree of suppression of Sertoli and Leydig cell number, on the one hand, and in blood levels of their secreted hormones, on the other hand, suggests that the hormone-producing function of these cells is normal and that it is the reduction in cell number that accounts for the reduction in hormone levels in blood. Three other pieces of information reinforce this view. First, average Leydig cell cytoplasmic volume was not reduced in T animals, and steroidogenic function of the Leydig cells invariably goes hand-in-hand with their cytoplasmic volume (Ewing and Zirkin 1983). Second, immunoexpression of functional markers (AMH in Sertoli cells, P450scc in Leydig cells) did not reveal any obvious difference between T and C testes. Third, there was no change in the hormonal drive (LH, FSH) to the T testes, compared with C testes, that could account for either the reductions in cell number or reduced hormone production.

Several pieces of new information suggest that testosterone plays an important role in regulating Sertoli cell proliferation in fetal (Johnston et al. 2004) and early postnatal (Atanassova et al. 2005; Johnston et al. 2004; Ramaswamy et al. 2000) life, raising the possibility that treatment-induced suppression of testosterone levels in the present study could have contributed to the reduction in Sertoli cell number. The observation of a reduction in intensity of AR immunoexpression in testes of treated animals, which can occur when testosterone levels are low and which leads to destabilization of the AR protein (Bremner et al. 1994; Zhou et al. 1995), provides indirect support for this possibility, as does the reduction in SMA immunostaining in peritubular cells. The perinatal effect of androgens on Sertoli cell number is considered to be mediated via the peritubular myoid cells that express ARs (Atanassova et al. 2005; Sharpe 2005), whereas Sertoli cells do not express ARs in fetal life (Sharpe 2005), as confirmed for the sheep in the present study. If the reduction in Sertoli cell number in T testes in the present study results secondarily from reduced testosterone levels/action, the primary adverse change in treated animals could be in the proliferation and/or differentiation of the Leydig cells.

In the present studies, male T fetuses showed growth restriction compared with controls of the same gestational age, whereas growth restriction was somewhat less evident in female fetuses from the same pregnancies and the treated pregnant ewes themselves did not show any difference in body weight from control ewes. The latter observation makes it unlikely that the growth effects observed in the male fetuses result simply from generalized toxicity. The reduction in body weight in male T fetuses essentially resulted in obliteration of the normal sex difference in body weight—a change associated with reduced testosterone levels in the treated males.

It is well established for both sheep (Mitchell et al. 2002) and humans (Hindmarsh et al. 2002; Schwarzler et al. 2004) that male fetuses grow at a higher rate on average than do female fetuses, such that males are larger for most growth parameters than are females at most gestational ages, as well as at birth. Testosterone production by the male may contribute to this difference (de Zegher et al. 1999; Gill and Hosking 1995). Whether or not the treatment-induced growth restriction in the male fetuses contributed to the reduction in testicular parameters in the present study is uncertain (see Rhind et al. 2001). Experimental growth restriction in fetal sheep, as a result of underfeeding of the pregnant ewes, was shown in one study to result in a 20% reduction in Sertoli cell number at around birth (Bielli et al. 2002), whereas three other studies found no significant change in Sertoli cell number and/or testis weight (Da Silva et al. 2003; Rae et al. 2002a, 2002b). Furthermore, in one of these studies (Rae et al. 2002b) an increase, rather than a decrease, in blood testosterone levels in growth-restricted male fetuses was reported, in contrast to the present findings. We are not aware of any published experimental manipulation in sheep that results in such major reductions in fetal testis weight and testicular hormones as found in treated animals in the present study. Irrespective of the exact interrelationships between body weight, testicular growth, and testosterone production, the present findings may be consistent with observations in the human that show a robust relationship between fetal growth restriction and increased risk of reproductive developmental disorders such as cryptorchidism and hypospadias (Akre et al. 1999; Weidner et al. 1999), testicular cancer (Skakkebaek et al. 2001; Toppari et al. 1996), and, less consistently, reduced sperm counts (Cigognani et al. 2002; Francois et al. 1997; Olsen et al. 2000). It is also of relevance that all of these disorders can be associated with reduced production or action of testosterone by the fetal testis (Sharpe and Skakkebaek 2003; Skakkebaek et al. 2001). It will be important to explore whether or not such abnormalities occur also in treated sheep in follow-up studies to those presently reported.

The present study does not identify the cause(s) of growth restriction and impaired testis development in treated male fetuses other than to demonstrate its association with the experimental application of sewage sludge to the pasture. The sewage sludge contains a complex cocktail of chemicals that includes heavy metals, alkylphenolic compounds, phthalates, and other classes of EDs (Rhind et al. 2002; Smith 1995), and cases could be made for several of these individual compounds playing some part in the presently observed changes in treated animals (see IPCS 2002; Sharpe and Irvine 2004; Sweeney et al. 2000; Toppari et al. 1996). So far, no major difference has been observed in phthalate or alkylphenol tissue levels in T and C ewes (Rhind et al. 2005b) and/or their fetuses, although some differences in heavy metal exposure are evident (Rhind et al. 2005a; Wilkinson et al. 2001). It seems most likely that the presently observed effects in male fetuses stem from exposure of their mothers to a combination of chemicals that were present in the sewage sludge, but identifying what these may be is a difficult task.

It is not possible, from the information available, to gauge whether the present findings in sheep have direct relevance to humans in terms of their exposure to such chemicals, because the effective chemicals present in sewage sludge have not been identified. Nevertheless, because human waste is an important contributor to sewage sludge, it is not unreasonable to assume that humans are themselves exposed to many of its constituent chemicals, even if the actual levels of exposure may be much lower in the human than was the case in sheep from the present studies, in which sewage sludge was delivered onto the surface of the soil or herbage to maximize ewe exposure. The levels of exposure achieved experimentally in this study also probably exceed those in animals that are grazing on land fertilized by sewage sludge according to current recommendations in the United Kingdom. However, the importance of the present study is that it demonstrates that prolonged exposure of ewes to a cocktail of chemicals present in sewage sludge, with clear relevance to real-world chemical exposures, retards fetal growth and leads to major attenuation of testicular growth and parallel attenuation of hormone production, and that the effects observed in the fetal sheep appear relevant to concerns about human male reproductive disorders that stem from maldevelopment of the fetal testis (Skakkebaek et al. 2001).

## Figures and Tables

**Table 1:** Antibodies used for immunohistochemistry.

Antibody	Type	Source	Dilution
AMH	Goat IgG	Santa Cruz Biotechnology (Santa Cruz, CA, USA)	1:1,000
AR	Rabbit IgG	Novocastra (Newcastle-Upon-Tyne, UK)	1:20
P450scc	Rabbit IgG	Chemicon (Hampshire, UK)	1:200
Ki-67	Rabbit IgG	DAKO (High Wycombe, UK)	1:100
SMA	Mouse monoclonal antibody	Sigma (Poole, UK)	1:3,000

**Table 2:** Effects of sewage sludge exposure on maternal and fetal body weight and on fetal testis weight (at GD110).

			Fetus			
Fetus no.	Treatment	Ewe weight (kg)	BW (g)	TW (mg)	TW:BW (%)	Birth status
F31	Control	90	2,224	478	2.1	Triplet
F7	Control	75	1,706	273	1.6	Twin
F10	Control	75	1,575	347	2.2	Twin
F13	Control	79	1,613	307	1.9	Twin
F22^a^	Control	84	2,733	366	1.3	Twin
F23^a^	Control	84	3,545	600	1.7	Twin
F29	Control	92	1,347	172	1.3	Quad
F30	Control	92	1,478	221	1.5	Quad
F33	Control	90	2,127	336	1.6	Triplet
F34	Control	93	2,053	346	1.7	Single
F39^a^	Control	81	3,059	505	1.7	Twin
F40^a^	Control	81	3,389	641	1.9	Twin
Mean ± SD		84.7 ± 6.6	2,237 ± 768	383 ± 145	1.7 ± 0.3	
Omitting F22, 23, 39, 40		84.9 ± 8.2	1,765 ± 326	310 ± 93	1.7 ± 0.3	
F1	Treated	80	1,979	204	1.0	Triplet
F2	Treated	80	1,678	190	1.1	Triplet
F5	Treated	78	2,175	302	1.4	Single
F11	Treated	85	1,435	218	1.5	Twin
F16	Treated	95	1,871	269	1.4	Twin
F19	Treated	80	1,246	197	1.6	Triplet
F21	Treated	80	1,082	138	1.3	Triplet
F25	Treated	75	1,053	192	1.8	Triplet
F26	Treated	75	1,021	165	1.6	Triplet
F38	Treated	81	1,466	269	1.8	Twin
F43	Treated	88	1,471	189	1.3	Triplet
F47^a^	Treated	66	789	152	1.9	Single
Mean ± SD		80.3 ± 7.9	1,438 ± 425	207 ± 50	1.5 ± 0.3	
Omitting F47		81.9 ± 6.5	1,497 ± 391	212 ± 49	1.4 ± 0.3	
Including all samples		NS	*p* = 0.0029	*p* = 0.0007	*p* = 0.037	
Omitting F22, 23, 39, 40, 47		NS	*p* = 0.06	*p* = 0.011	*p* = 0.0286	

Abbreviations: BW, body weight; NS, not significant; TW, testis weight.

a Data for animals that may differ from the rest in terms of gestational age (see “Results”).
